# Trauma and Early Puberty May Be Stronger Predictors of Early Tobacco Initiation in Girls Compared to Boys

**DOI:** 10.31586/jbls.2025.1135

**Published:** 2025-01-23

**Authors:** Shervin Assari, Hossein Zare

**Affiliations:** 1 Department of Internal Medicine, Charles R. Drew University of Medicine and Science, Los Angeles, CA, United States; 2 Department of Family Medicine, Charles R. Drew University of Medicine and Science, Los Angeles, CA, United States; 3 Department of Urban Public Health, Charles R. Drew University of Medicine and Science, Los Angeles, CA, United States; 4 Marginalization-Related Diminished Returns (MDRs) Center, Los Angeles, CA, United States; 5 Department of Health Policy and Management, Johns Hopkins Bloomberg School of Public Health, Baltimore, MD, United States; 6 School of Business, University of Maryland Global Campus (UMGC), Adelphi, MD, United States

**Keywords:** Socioeconomic status, Trauma, Early Puberty, Tobacco Use, Sex Differences, ABCD Study, Structural Equation Modeling

## Abstract

**Objective:**

This study investigates the pathways linking socioeconomic status (SES), trauma, early puberty, and tobacco use, with a focus on how these relationships differ by sex. Using data from the Adolescent Brain Cognitive Development (ABCD) study, we explore how SES and race contribute to trauma exposure, which in turn may influence early puberty and tobacco use. The study also examines potential mediating effects of trauma and early puberty on the association between SES and tobacco use, while comparing these pathways for males and females.

**Methods:**

Data were drawn from the ABCD study, and structural equation modeling (SEM) was employed to test direct and indirect pathways between SES, trauma, early puberty, and tobacco use. The sample was stratified by sex to assess differences in these relationships for males and females. Key predictors included SES, race, and age, while outcomes were trauma, early puberty, and tobacco use. The model assessed mediating effects of trauma and early puberty on tobacco use.

**Results:**

Trauma was a significant predictor of early puberty for females (B = 0.032, SE = 0.015, p = 0.039) but not males. Early puberty was significantly linked to tobacco use for females (B = 0.048, SE = 0.015, p = 0.001) but not for males. Additionally, trauma had an effect on tobacco use among females (B = 0.048, SE = 0.014, p < 0.001) but not males. Lower SES was significantly associated with higher trauma exposure for both males (B = −0.109, SE = 0.014, p < 0.001) and females (B = −0.110, SE = 0.015, p < 0.001).

**Conclusions:**

The findings suggest that trauma and early puberty play more significant roles in the pathways from SES to tobacco use for females than for males. While trauma and early puberty are crucial mediators for females, these factors are less predictive for males. These results highlight the importance of sex-specific interventions targeting trauma and early puberty as pathways to early tobacco use.

## 1. Introduction

Early tobacco use is a significant public health concern due to its association with long-term negative health outcomes, including addiction, respiratory diseases, and cardiovascular problems [1,2]. Identifying the early-life factors that contribute to tobacco use is essential for developing targeted prevention and intervention strategies. Social determinants of health, such as socioeconomic status (SES), are central in shaping individuals’ exposure to various stressors, such as trauma, that can influence behavioral outcomes like tobacco use [3].

Trauma may act as a critical mediator in the relationship between SES and tobacco use among children and adolescents [3]. Low SES often exposes youth to higher levels of adversity, including community violence, household instability, and chronic stress. These experiences accumulate as trauma, which affects both psychological and physiological development. Children from lower-SES families may turn to tobacco as a maladaptive coping mechanism to manage stress and trauma, particularly in environments with limited access to mental health resources or healthy outlets for stress. For low SES youth, who are more likely to experience poverty and trauma, this pathway may be especially pronounced, further exacerbating health disparities in early tobacco use [4].

Another potential mechanism linking low SES to early tobacco use is early puberty [5,6]. Research suggests that stress and trauma can accelerate biological maturation, including the onset of puberty, which is associated with an increased likelihood of engaging in risky behaviors, such as substance use. Early puberty may serve as a biological marker of the body’s stress response, with accelerated maturation representing an adaptation to environmental adversity [7]. This premature initiation of risk behaviors often occurs before youth have developed the cognitive and emotional capacities to handle these choices responsibly. Moreover, youth from low-SES backgrounds, already at greater risk for trauma, may be doubly impacted by the combined effects of early puberty and stress.

Sex differences may also play an important role in how SES and trauma influence the pathway to tobacco use [8]. Research suggests that the effects of trauma may vary for males and females, with females potentially experiencing more pronounced impacts of trauma on behavioral outcomes. Boys and girls may be differently vulnerable to the effects of trauma on early puberty, which can increase their risk of engaging in tobacco use. In contrast, boys may exhibit different responses to trauma, possibly engaging in other forms of risk behavior that diverge from substance use. Understanding these sex-specific mechanisms is crucial for designing interventions that are sensitive to both gender and social context.

Despite the significance of these pathways, few studies have thoroughly examined the complex mechanisms linking SES, trauma, early puberty, and tobacco use, particularly in light of sex and racial differences. Such research is critical for identifying intervention points that can help mitigate early tobacco use and inform targeted prevention strategies. Structural Equation Modeling (SEM) provides a robust approach to analyzing these relationships, enabling the exploration of both direct and indirect pathways. Through SEM, we can assess how SES influences trauma, how trauma mediates the relationship between SES and early puberty, and how these factors, in turn, contribute to tobacco use. Moreover, SEM allows for the examination of potential sex differences, illuminating how the pathways may differ for males and females.

This study leverages data from the Adolescent Brain Cognitive Development (ABCD) study to examine the intersections of SES, trauma, early puberty, and tobacco use. Using SEM, we aim to explore these complex pathways and contribute to a deeper understanding of how social and biological processes interact to shape health disparities in early adolescence. Specifically, we investigate the roles of race and SES in modifying these pathways, while also examining potential sex differences. Our findings will help identify critical intervention points for reducing early tobacco use among high-risk groups, particularly those facing compounded risks due to low SES, racial disparities, and gendered responses to trauma.

## 2. Methods

### 2.1. Settings and Design

This study utilized data from the Adolescent Brain Cognitive Development (ABCD) Study, a large-scale, longitudinal research initiative designed to examine brain development and child health in the United States. The ABCD study recruited over 11,000 children aged 9–10 years from 21 sites across the country, using a multi-stage probability sampling method to ensure a diverse and representative sample. Data collection involved a combination of neuroimaging, behavioral assessments, and questionnaires completed by both children and their parents. The current analysis leverages cross-sectional baseline data from the ABCD study, focusing on brain structure, socioeconomic factors, and demographic variables.

### 2.2. Sample and Sampling

The initial sample for the ABCD study included 11,878 children. For this analysis, we restricted the sample to children identified by their parents as either Black or White, consistent with our focus on racial disparities. To ensure reliable estimates of brain structure, children with missing or poor-quality neuroimaging data were excluded from the sample.

### 2.3. Measures

#### Outcome (Tobacco Use Initiation):

Tobacco use initiation was defined as the first reported use of any tobacco product, including cigarettes, e-cigarettes, and other tobacco-related products, at any follow-up assessment during the study period. Annual assessments involved the iSay Sipping Inventory for recent or first experimentation with nicotine products. Follow-up questions on circumstances surrounding first use were administered at one-time point, however, such information was not included in our analysis. At Baseline (Y0), youth reported lifetime use of tobacco products with a web-based Timeline Follow-Back (TLFB) interview for substances used in the past six months (only for baseline evaluation at time 0) or since the last study session (for measures at months six, twelve, eighteen, twenty-four, and thirty months). The analysis covered various substances, and Mid-Year phone follow-ups contributed to a comprehensive past-year tobacco use for each yearly follow-up. For current analyses, tobacco use variables were defined as follows: Tobacco use experimentation as low-level tobacco use (e.g., puffing). Tobacco initiation as reporting > 1 puff of nicotine. Tobacco use onset as the time of reported tobacco use other than experimentation.

#### Mediator 1 (Trauma):

Parents were interviewed regarding the trauma experienced by the child. The Kiddie Schedule for Affective Disorders and Schizophrenia (K-SADS) was used to measure trauma. This is a semi-structured interview aimed at the early detection of high-risk youth. The items included: (1) “A car accident in which your child or another person in the car was hurt bad enough to require medical attention”, (2) “Another significant accident for which your child needed specialized and intensive medical treatment”, (3) “Witnessed or caught in a fire that caused significant property damage or personal injury”, (4) “Witnessed or caught in a natural disaster that caused significant property damage or personal injury”, (5) “Witnessed or present during an act of terrorism (e.g., Boston marathon bombing)”, (6) “Witnessed death or mass destruction in a war zone”, (7) “Witnessed someone shot or stabbed in the community”, (8) “Shot, stabbed, or beaten brutally by a non-family member”, (9) “Shot, stabbed, or beaten brutally by a grown up in the home”, (10) “Beaten to the point of having bruises by a grown up in the home”, (11) “A non-family member threatened to kill your child”, (12) “A family member threatened to kill your child”, (13) “Witness the grownups in the home push, shove or hit one another”, (14) “A grown up in the home touched your child in his or her privates, had your child touch their privates, or did other sexual things to your child”, (15) “An adult outside your family touched your child in his or her privates, had your child touch their privates or did other sexual things to your child”, (16) “A peer forced your child to do something sexually”, and (17) “Learned about the sudden unexpected death of a loved one”. Response items for each item were 0 (no) or 1 (yes). We counted the number of traumatic events, and, given the extreme skewness of the count of traumatic events, we calculated a variable as zero traumatic events, one traumatic event, and two or more traumatic events (Cronbach’s alpha = 0.637).

#### Mediator 2 (Early Puberty):

Pubertal development was assessed using the Pubertal Development Scale (PDS), a questionnaire designed for self or parent rating that closely resembles traditional Tanner staging without relying on reference images. This tool evaluates the physical secondary sexual characteristics associated with puberty through ordinal ratings. The PDS includes seven items: three are neutral regarding sex and measure changes in skin, body hair, and height growth. Additionally, there are two items tailored for females—breast development and the onset of menarche (the first menstrual period)—and two specifics to males—voice changes and facial hair growth. Each item is scored on a scale from 1 to 4, where 1 indicates no development has started, 2 suggests minimal development, 3 denotes that development is clearly in progress, and 4 signifies that development is complete. Menarche is assessed with a binary response (yes/no). The PDS demonstrates strong inter-rater reliability between parent and self-assessments compared to clinical evaluations and shows a significant correlation with plasma levels of gonadal hormones. For our analysis, we utilized parent-rated development scores, as these have consistently been found to align more closely with trained clinician assessments than child-rated scores. We created a binary variable indicating the presence of any signs of puberty versus no signs at the baseline measurement.

#### Predictor (Family SES):

The key independent variable was family SES, defined based on parental education, household income, and marital status of the family. *Parental Educational Attainment*. Participants were asked, “What is the highest grade or level of school you have completed or the highest degree you have received?” Responses were 0 = Never attended/Kindergarten only; 1 = 1st grade; 2 = 2nd grade; 3 = 3rd grade; 4 = 4th grade 4; 5 = 5th grade; 6 = 6th grade 6; 7 = 7th grade 7; 8 = 8th grade; 9 = 9th grade; 10 = 10th grade 10; 11 = 11th grade; 12 = 12th grade; 13 = high school graduate; 14 = GED or equivalent diploma; 15 = some college; 16 = associate degree: occupational; 17 = associate degree: academic program; 18 = bachelor’s degree (ex. BA; 19 = master’s degree (ex. MA; 20 = professional school degree (ex. MD; 21 = doctoral degree. This variable was an interval measure with a range between 1 and 21, with a higher score indicating higher educational attainment. Family income was a continuous measure ranging from 1 to 10, with a higher score indicating higher income. The exact question was, “What is your total combined family income for the past 12 months? This should include income (before taxes and deductions) from all sources, wages, rent from properties, social security, disability and veteran’s benefits, unemployment benefits, workman”. Responses included 1 = Less than $5000; 2 = $5000; 3 = $12,000; 4 = $16,000; 5 = $25,000; 6 = $35,000; 7 = $50,000; 8 = $75,000; 9 = $100,000; 10 = $200,000. Marital status of the family was self-reported by the parent and was coded 0 for unwed/divorced/separated and 1 for married. This SES variable was treated as a 0 vs 1 categorical variable, with 1 representing higher SES.

### 2.4. Covariates

We controlled for several demographic variables that could influence brain development, including the child’s age, sex, and race. Race was self-identified by the parent as either White, Black, and other. Age and sex were included to account for normal developmental differences in cortical volume across children.

### 2.5. Statistical Analysis

We employed Structural Equation Modeling (SEM) to test the associations between family SES (a dichotomous measure) and early tobacco initiation (a dichotomous measure), with trauma (n) and early puberty (a dichotomous measure) as a potential mediators and sex as a moderator. Our analyses were conducted using SEM in Stata, and we used maximum likelihood estimation to account for missing data. Model fit was assessed using standard indices such as the Comparative Fit Index (CFI) and Root Mean Square Error of Approximation (RMSEA). Significance was evaluated at p < .05.

### 2.6. Ethics

The ABCD study received approval from the Institutional Review Boards (IRBs) at each of the 21 data collection sites. Informed consent was obtained from all parents or legal guardians, and assent was obtained from children before participation. This study’s secondary analysis of de-identified ABCD data was approved by [Your Institution’s IRB], ensuring compliance with ethical guidelines for human subject’s research.

## 3. Results

The results of the structural equation model (SEM) are presented in Table 1, summarizing the direct effects of age, sex, socioeconomic status (SES), race, trauma, and early puberty on trauma, early puberty, and tobacco use outcomes. The SEM results highlight important pathways linking SES, race, trauma, early puberty, and tobacco use. Lower SES and being Black were associated with higher trauma exposure, which in turn predicted early puberty and increased tobacco use. Early puberty also independently contributed to higher tobacco use. Notably, Black youth showed lower rates of tobacco use despite being at greater risk for trauma and early puberty, suggesting potential protective factors or differing mechanisms in the path from trauma and early puberty to tobacco use for this group. These findings emphasize the complex interplay of social, biological, and racial factors in shaping early tobacco use behaviors.

Trauma SES and race were both significant predictors of trauma. Lower SES was associated with higher trauma exposure (B = −0.109, SE = 0.010, p < 0.001), indicating that youth from lower socioeconomic backgrounds experienced more trauma. In addition, being Black was associated with increased trauma (B = 0.036, SE = 0.010, p < 0.001), suggesting that Black youth experienced heightened trauma relative to their peers. Age (B = −0.002, SE = 0.009, p = 0.864) and male gender (B = 0.000, SE = 0.009, p = 0.975) were not significant predictors of trauma.

Early Puberty Trauma had a significant positive association with early puberty (B = 0.023, SE = 0.010, p = 0.025), suggesting that youth exposed to more trauma were more likely to experience early pubertal onset. SES was negatively associated with early puberty (B = −0.035, SE = 0.012, p = 0.003), indicating that lower-SES youth experienced earlier puberty. Additionally, being Black was associated with earlier pubertal onset (B = 0.087, SE = 0.012, p < 0.001), while male gender was negatively associated with early puberty (B = −0.079, SE = 0.010, p < 0.001), with females experiencing earlier puberty. Age was also a significant predictor of early puberty (B = 0.112, SE = 0.010, p < 0.001), as expected given the natural progression of biological maturation.

Tobacco Use Trauma (B = 0.035, SE = 0.009, p < 0.001) and early puberty (B = 0.033, SE = 0.010, p = 0.001) were both significant predictors of tobacco use, suggesting that youth who experienced more trauma or who underwent early puberty were more likely to initiate tobacco use. SES was negatively associated with tobacco use (B = −0.072, SE = 0.011, p < 0.001), indicating that lower-SES youth were more likely to use tobacco. Interestingly, being Black was associated with reduced tobacco use (B = −0.036, SE = 0.011, p = 0.001), despite Black youth having higher trauma exposure and earlier puberty. Age was positively associated with tobacco use (B = 0.070, SE = 0.009, p < 0.001), while gender was not a significant predictor (B = −0.011, SE = 0.009, p = 0.232).

### 3.1. Results by Sex

Table 2 shows the results of the structural equation model (SEM) for males and females highlight several key differences in the significant pathways linking socioeconomic status (SES), trauma, early puberty, and tobacco use. Below, we focus on paths that are significant for one sex but not the other, showcasing how these relationships differ between males and females.

### 3.2. Trauma

#### Race (Black) → Trauma:

Race had a significant positive association with trauma exposure for males (B = 0.044, SE = 0.014, p = 0.002), indicating that Black males experienced higher levels of trauma than their peers. This effect was not significant for females (B = 0.027, SE = 0.015, p = 0.079), suggesting that the link between race and trauma is stronger in males.

### 3.3. Early Puberty

#### Trauma → Early Puberty:

For females, trauma was significantly associated with early puberty (B = 0.032, SE = 0.015, p = 0.039), indicating that females who experienced more trauma were more likely to undergo early puberty. This relationship was not significant for males (B = 0.020, SE = 0.014, p = 0.171), suggesting a stronger impact of trauma on biological maturation in females.

#### Age → Early Puberty:

Age was a stronger predictor of early puberty for females (B = 0.181, SE = 0.015, p < 0.001) compared to males (B = 0.064, SE = 0.014, p < 0.001), with females showing a steeper age-related progression toward puberty.

### 3.4. Tobacco Use

#### Trauma → Tobacco Use:

Trauma was significantly associated with tobacco use for females (B = 0.048, SE = 0.014, p < 0.001), but this path was not significant for males (B = 0.024, SE = 0.013, p = 0.063). This finding suggests that females may be more likely than males to engage in tobacco use as a response to trauma.

#### Early Puberty → Tobacco Use:

Early puberty had a significant positive effect on tobacco use for females (B = 0.048, SE = 0.015, p = 0.001), whereas this path was not significant for males (B = 0.021, SE = 0.014, p = 0.118). This indicates that early pubertal timing is more strongly linked to tobacco use among females than males.

#### Race (Black) → Tobacco Use:

Being Black was associated with lower tobacco use for females (B = −0.052, SE = 0.015, p = 0.001), but this association was not significant for males (B = −0.021, SE = 0.015, p = 0.155). This suggests that Black females may have protective factors that reduce tobacco use, a pattern not observed among Black males.

### 3.5. Summary

These sex-specific findings highlight key differences in how trauma and early puberty influence tobacco use between males and females. Trauma appears to have a stronger effect on early puberty and tobacco use for females, while race is a more significant predictor of trauma for males. Additionally, early puberty plays a more substantial role in predicting tobacco use among females. These distinctions underscore the need for tailored interventions that address gender-specific pathways to early tobacco use.

## 4. Discussion

This study investigates the pathways linking socioeconomic status (SES), trauma, early puberty, and tobacco use, with a focus on how these relationships differ by sex. Using data from the Adolescent Brain Cognitive Development (ABCD) study, we explore how SES and race contribute to trauma exposure, which in turn may influence early puberty and tobacco use. The study also examines potential mediating effects of trauma and early puberty on the association between SES and tobacco use, while comparing these pathways for males and females.

In the overall sample, we found that high socioeconomic status (SES) predicts tobacco use through higher trauma, which in turn predicts early puberty. In this view, trauma and puberty act as serial mediators in the relationship between SES and tobacco use. The effects of SES on tobacco use have been well-documented in the literature, indicating that higher SES often correlates with better access to resources, including education, mental health support, and recreational activities, which can mitigate the adverse effects of stressors. Conversely, lower SES is associated with increased exposure to environmental stressors and trauma, leading to a higher likelihood of engaging in risky behaviors, including tobacco use. Studies have shown that trauma can precipitate maladaptive coping mechanisms, such as substance use, as individuals seek relief from psychological distress. Additionally, early puberty has been linked to increased susceptibility to substance use, as it can disrupt typical developmental trajectories, leading adolescents to adopt adult-like behaviors prematurely. The interplay of these factors highlights the complex mechanisms through which SES influences health outcomes, underscoring the need for targeted interventions that address both trauma and developmental processes.

Low SES contributes to early puberty through various interrelated mechanisms. Children from low-SES backgrounds often face chronic stressors, including financial instability, family conflict, and exposure to violence, which can trigger physiological stress responses. Research indicates that sustained exposure to such adversities can accelerate biological maturation, including the onset of puberty. Moreover, lower SES is often linked to inadequate access to healthcare and mental health resources, which can exacerbate the impact of trauma and hinder coping strategies. Additionally, environmental factors, such as living in high-crime neighborhoods or experiencing food insecurity, can further compound stress and trauma, leading to earlier pubertal onset. This intersection of socioeconomic disadvantage and psychological stressors creates a cycle of adversity that not only impacts biological development but also increases the risk of engaging in risky behaviors, such as tobacco use, during adolescence. Thus, addressing the multifaceted nature of low SES is crucial for understanding and intervening in the pathways leading to early puberty and associated health risks.

However, while this mechanism may have provided adaptive advantages in ancestral environments, the implications for modern youth, particularly those from low socioeconomic backgrounds, can be detrimental. Early maturation is associated with a range of negative outcomes, including increased susceptibility to risky behaviors such as substance use, as adolescents may not have fully developed the cognitive and emotional skills needed to navigate adult responsibilities effectively. Thus, while early maturation may have evolved as a survival strategy, it can lead to complex health risks and behavioral challenges in contemporary settings, particularly among those facing chronic stress and trauma. Understanding these dynamics can help inform interventions aimed at mitigating the adverse effects of early maturation and promoting healthier developmental trajectories for at-risk youth.

The results from the SEM reveal significant sex differences in the pathways linking SES, trauma, early puberty, and tobacco use. These findings underscore the complexity of adolescent substance use and the necessity of considering gender-specific factors in the analysis. The pathways that are significant for one sex but not the other highlight the distinct experiences and developmental trajectories of males and females in relation to these variables.

One notable finding is the strong positive association between race (specifically being Black) and trauma exposure among males, which was not observed for females. This suggests that Black males experience higher levels of trauma compared to their peers, potentially due to the unique social and environmental challenges they face. Literature supports this observation, indicating that racial minorities, particularly males, often encounter stressors such as discrimination and violence, which can contribute to increased trauma exposure. The absence of a significant relationship for females may indicate that the factors leading to trauma are experienced differently across genders, suggesting that interventions targeting trauma for Black males might need to be more intensive and context-specific to address their unique challenges effectively.

In terms of the impact of trauma on early puberty, the results indicate that trauma significantly influences early puberty in females but not in males. This finding aligns with existing literature that suggests girls may be more sensitive to psychosocial stressors, which can accelerate biological maturation. Girls exposed to high levels of trauma may undergo early puberty as a response to their stressful environments, whereas boys might not exhibit the same sensitivity. The steeper age-related progression toward early puberty in females further emphasizes this point and suggests that interventions aimed at mitigating trauma’s effects may be particularly critical for girls.

When examining the pathways to tobacco use, the results show that trauma has a significant association with tobacco use for females, while this relationship was not significant for males. This indicates that females may be more likely to use tobacco as a coping mechanism in response to trauma. This aligns with literature suggesting that females are more likely to engage in substance use as a means of dealing with psychological distress. Conversely, the lack of a significant relationship for males suggests that their coping mechanisms may differ, potentially relying more on externalizing behaviors rather than substance use.

Additionally, early puberty significantly predicted tobacco use among females but not among males. This finding highlights the importance of early pubertal timing as a risk factor for tobacco use in females, reinforcing literature that indicates girls who experience early puberty are more likely to engage in risk behaviors, including substance use. The fact that this association is not significant for males suggests that early maturation may not carry the same implications for substance use among boys, pointing to the need for tailored interventions that consider these gender-specific pathways.

Interestingly, the influence of race on tobacco use also varied between sexes. For females, being Black was associated with lower tobacco use, suggesting the presence of protective factors that mitigate the risk of tobacco use in this demographic. This contrasts with findings for males, where the relationship was not significant. Literature indicates that cultural factors, community support, and resilience may contribute to lower rates of tobacco use among Black females, highlighting the need for further exploration of these protective factors.

### 4.1. Limitations

This study has several limitations that should be acknowledged. First, our measures of SES, trauma, and puberty were cross-sectional, which limits our ability to draw causal inferences about the relationships between changes in SES, trauma, pubertal timing, and tobacco use. Longitudinal assessments of SES, trauma, and puberty trajectories would offer stronger evidence regarding temporal relationships and potential causal pathways. Second, although structural equation modeling (SEM) allows for the examination of complex relationships, it relies on underlying assumptions that may not fully capture the intricacies of the data. Additionally, the measures of trauma, SES, and tobacco use are based on self-reports, which may introduce biases and affect the validity of the findings. Third, the study’s focus on a specific age range within the ABCD study may limit the generalizability of the results to other age groups or populations. Finally, the analysis did not account for all potential confounding variables, such as family dynamics or peer influences, which may also contribute to the observed relationships.

### 4.2. Future Research

Future research should aim to address these limitations by employing longitudinal designs that can better elucidate causal pathways and the dynamic interplay between SES, trauma, early puberty, and tobacco use over time. Additionally, qualitative studies exploring the lived experiences of adolescents could provide deeper insights into the context and mechanisms behind these relationships. Research should also investigate the role of protective factors, such as community support and resilience, that may buffer the negative effects of trauma and low SES on tobacco use, particularly among different racial and ethnic groups. Expanding the sample to include more diverse populations can enhance the understanding of how cultural and contextual factors influence these pathways. Finally, examining gender differences in coping mechanisms and substance use could provide valuable information for tailoring interventions.

### 4.3. Implications

The findings from this study have significant implications for public health and intervention strategies aimed at reducing early tobacco use among adolescents. Recognizing the distinct pathways through which trauma and early puberty influence tobacco use in males and females can inform the development of targeted prevention programs. For females, interventions that address trauma and its psychological impacts may be critical in preventing early tobacco use. For males, programs that focus on the unique stressors related to race and trauma exposure may be necessary to mitigate their risk. Additionally, the findings suggest the need for culturally sensitive interventions that consider the protective factors present in different communities, particularly among Black youth. By addressing the specific needs and experiences of adolescents based on their sex, race, and socioeconomic context, health practitioners and policymakers can develop more effective strategies to combat early tobacco use and ultimately reduce health disparities among at-risk populations.

## 5. Conclusion

In summary, the findings emphasize critical differences in how trauma and early puberty influence tobacco use between males and females. The stronger effects of trauma on early puberty and tobacco use for females, along with the significant racial differences in trauma exposure for males, underscore the necessity of considering gender in research and interventions related to adolescent substance use. These distinctions point to the potential for developing targeted strategies that address the unique experiences and needs of both sexes, ultimately improving prevention efforts for early tobacco use among at-risk youth.

## Figures and Tables

**Figure 1.**
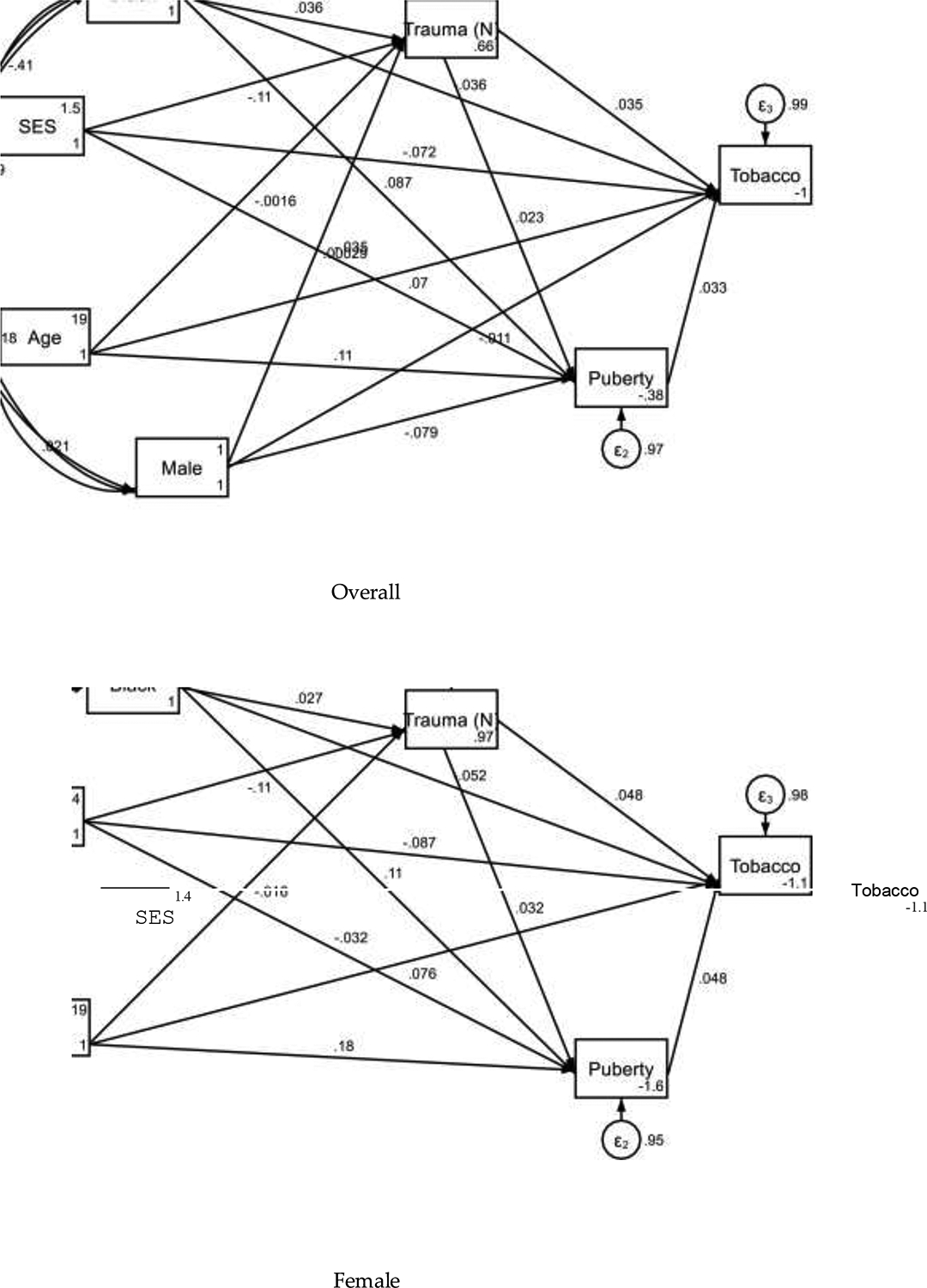

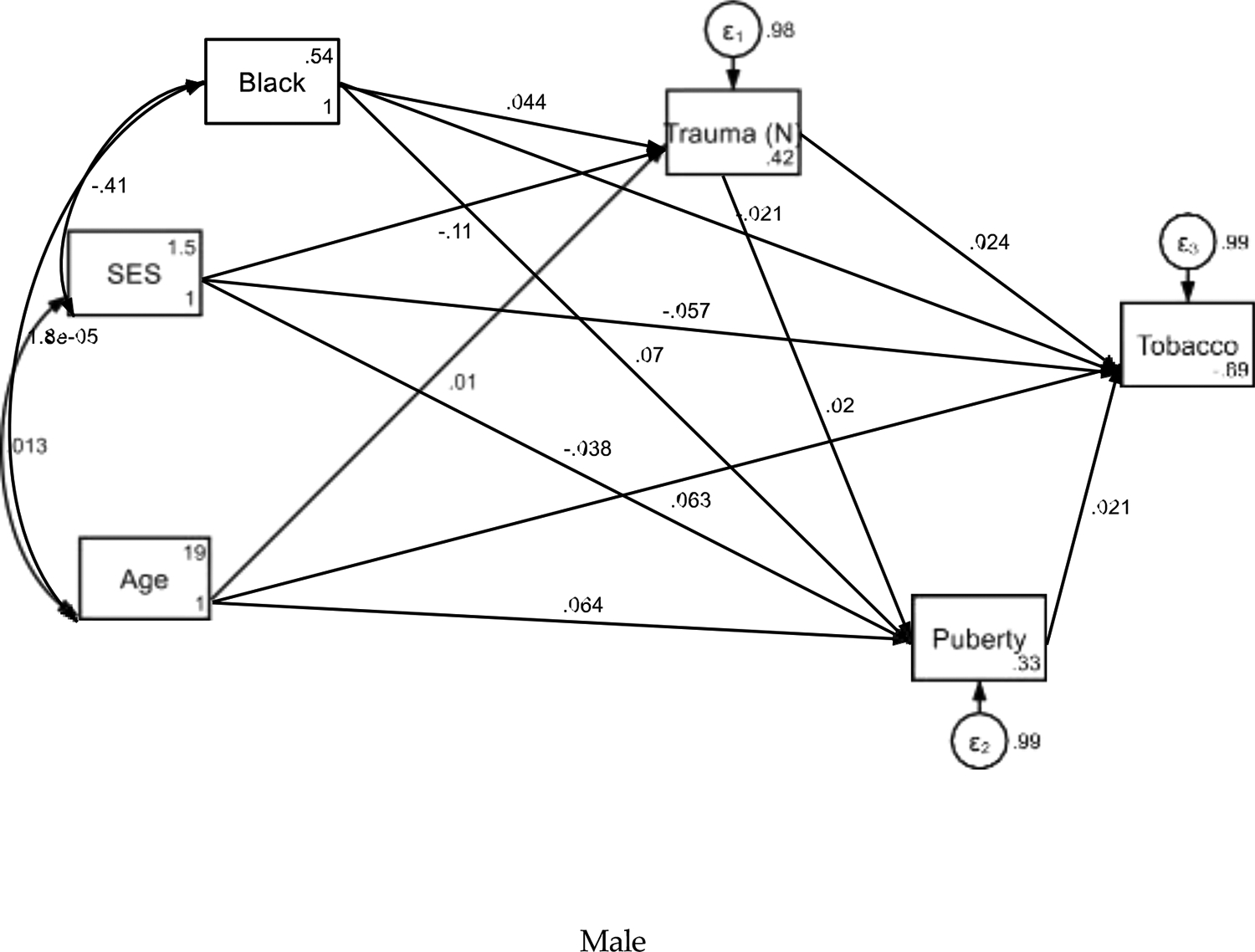
Summary of structural equation models overall and by sex

**Table 1.** Summary of structural equation model (SEM), overall

			B	SE	95%	CI	p
Age	→	Trauma	−0.002	0.009	−0.020	0.017	0.864
Male	→	Trauma	0.000	0.009	−0.018	0.018	0.975
Socioeconomic Status (SES)	→	Trauma	−0.109	0.010	−0.130	−0.089	< 0.001
Black	→	Trauma	0.036	0.010	0.015	0.056	< 0.001
Intercept	→	Trauma	0.662	0.174	0.320	1.003	< 0.001
Trauma	→	Early Puberty	0.023	0.010	0.003	0.044	0.025
Age	→	Early Puberty	0.112	0.010	0.092	0.131	< 0.001
Male	→	Early Puberty	−0.079	0.010	−0.099	−0.059	< 0.001
Socioeconomic Status (SES)	→	Early Puberty	−0.035	0.012	−0.058	−0.012	0.003
Black	→	Early Puberty	0.087	0.012	0.064	0.110	< 0.001
Intercept	→	Early Puberty	−0.376	0.192	−0.751	0.000	0.050
Trauma	→	Tobacco Use	0.035	0.009	0.016	0.053	< 0.001
Early Puberty	→	Tobacco Use	0.033	0.010	0.014	0.053	0.001
Age	→	Tobacco Use	0.070	0.009	0.052	0.088	< 0.001
Male	→	Tobacco Use	−0.011	0.009	−0.029	0.007	0.232
Socioeconomic Status (SES)	→	Tobacco Use	−0.072	0.011	−0.093	−0.051	< 0.001
Black	→	Tobacco Use	−0.036	0.011	−0.057	−0.015	0.001
Intercept	→	Tobacco Use	−1.013	0.172	−1.350	−0.675	< 0.001

**Table 2.** Summary of structural equation model (SEM), by sex

				Females					Males			
			B	SE	95%	CI	p	B	SE	95%	CI	p
Age	→	Trauma	−0.016	0.013	−0.042	0.010	0.229	0.010	0.013	−0.015	0.035	0.423
Socioeconomic Status (SES)	→	Trauma	−0.110	0.015	−0.139	−0.080	0.000	−0.109	0.014	−0.138	−0.081	0.000
Black	→	Trauma	0.027	0.015	−0.003	0.056	0.079	0.044	0.014	0.016	0.072	0.002
Intercept	→	Trauma	0.967	0.253	0.471	1.463	0.000	0.417	0.240	−0.054	0.888	0.083
Trauma	→	Early Puberty	0.032	0.015	0.002	0.062	0.039	0.020	0.014	−0.008	0.048	0.171
Age	→	Early Puberty	0.181	0.015	0.152	0.210	0.000	0.064	0.014	0.037	0.090	0.000
Socioeconomic Status (SES)	→	Early Puberty	−0.032	0.018	−0.066	0.003	0.072	−0.038	0.016	−0.069	−0.007	0.016
Black	→	Early Puberty	0.113	0.017	0.079	0.147	0.000	0.070	0.016	0.039	0.101	0.000
Intercept	→	Early Puberty	−1.623	0.284	−2.180	−1.067	0.000	0.332	0.257	−0.171	0.835	0.196
Trauma	→	Tobacco Use	0.048	0.014	0.021	0.075	0.000	0.024	0.013	−0.001	0.050	0.063
Early Puberty	→	Tobacco Use	0.048	0.015	0.019	0.077	0.001	0.021	0.014	−0.005	0.048	0.118
Age	→	Tobacco Use	0.076	0.014	0.050	0.102	0.000	0.063	0.013	0.038	0.088	0.000
Socioeconomic Status (SES)	→	Tobacco Use	−0.087	0.015	−0.118	−0.057	0.000	−0.057	0.015	−0.086	−0.028	0.000
Black	→	Tobacco Use	−0.052	0.015	−0.082	−0.022	0.001	−0.021	0.015	−0.050	0.008	0.155
Intercept	→	Tobacco Use	−1.137	0.251	−1.629	−0.645	0.000	−0.891	0.238	−1.358	−0.424	0.000
